# Heart in Acromegaly: The Echocardiographic Characteristics of Patients Diagnosed with Acromegaly in Various Stages of the Disease

**DOI:** 10.1155/2018/6935054

**Published:** 2018-07-11

**Authors:** Agata Popielarz-Grygalewicz, Jakub S. Gąsior, Aleksandra Konwicka, Paweł Grygalewicz, Maria Stelmachowska-Banaś, Wojciech Zgliczyński, Marek Dąbrowski

**Affiliations:** ^1^Cardiology Clinic of Physiotherapy Division of the 2nd Faculty of Medicine, Medical University of Warsaw, Warsaw, Poland; ^2^Faculty of Health Sciences and Physical Education, Kazimierz Pulaski University of Technology and Humanities in Radom, Radom, Poland; ^3^Department of Endocrinology, The Centre of Postgraduate Medical Education, Bielanski Hospital, Warsaw, Poland; ^4^Department of Cardiology, Bielanski Hospital, Warsaw, Poland

## Abstract

To determine whether the echocardiographic presentation allows for diagnosis of acromegalic cardiomyopathy. 140 patients with acromegaly underwent echocardiography as part of routine diagnostics. The results were compared with the control group comprising of 52 age- and sex-matched healthy volunteers. Patients with acromegaly presented with higher BMI, prevalence of arterial hypertension, and glucose metabolism disorders (i.e., diabetes and/or prediabetes). In patients with acromegaly, the following findings were detected: increased left atrial volume index, increased interventricular septum thickness, increased posterior wall thickness, and increased left ventricular mass index, accompanied by reduced diastolic function measured by the following parameters: E'med., E/E', and E/A. Additionally, they presented with abnormal right ventricular systolic pressure. All patients had normal systolic function measured by ejection fraction. However, the values of global longitudinal strain were slightly lower in patients than in the control group; the difference was statistically significant. There were no statistically significant differences in the size of the right and left ventricle, thickness of the right ventricular free wall, and indexed diameter of the ascending aorta between patients with acromegaly and healthy volunteers. None of 140 patients presented systolic dysfunction, which is the last phase of the so-called acromegalic cardiomyopathy. Some abnormal echocardiographic parameters found in acromegalic patients may be caused by concomitant diseases and not elevated levels of GH or IGF-1 alone. The potential role of demographic parameters like age, sex, and/or BMI requires further research.

## 1. Introduction

Acromegaly is a rare endocrine disorder, with a prevalence of approximately 50–70 per million. It is caused by excessive secretion of growth hormone (GH), typically secondary to a pituitary adenoma originating from somatotropic cells (>98% of cases) [1, 2]. The very rare cause of GH oversecretion is ectopic tumour [3].

Clinical manifestations depend on the levels of GH and insulin-like growth factor-1 (IGF-1), age, tumour size, and the time organs are exposed to elevated hormone levels (time between disease onset and diagnosis). The average time between the initial hormonal imbalance and disease diagnosis is estimated at approximately 10 years. Therefore, patients typically present to the endocrinologist when organs have been exposed to high levels of GH and IGF-1 for many years [4].

The symptoms resulting from the influence of GH and IGF-1 on the cardiovascular system are defined in the literature as “acromegalic cardiomyopathy” [5–7]. It has been established that this condition develops in three phases. In the first phase, hypertrophy and increased contractility of the myocardium of the left and right ventricle occur as a result of increased levels of GH and IGF-1. In the second phase, the elasticity of the ventricular wall reduces, which causes filling disorders and diastolic malfunction. The ventricular dilation and systolic dysfunction comprise the final phase of acromegalic cardiomyopathy [4, 8].

This study presents the echocardiographic characteristics of 140 patients with acromegaly to explore whether the term “acromegalic cardiomyopathy” is justified in relation to these cardiovascular manifestations. The aim of the study was to evaluate the morphological and functional parameters of the cardiovascular system based on echocardiography in patients with acromegaly and to determine whether the echocardiographic presentation allows for diagnosis of acromegalic cardiomyopathy.

## 2. Materials and Methods

### 2.1. Study Population

Between 2008 and 2016, we evaluated 140 patients diagnosed with acromegaly by means of echocardiography, as a routine diagnostic procedure. In the study group, 91 patients (65%) were recently diagnosed with acromegaly and had not received any treatment. Forty-nine patients (35%) had a long history of acromegaly (lasting for several years). Some patients were considered cured following radical neurosurgery, whereas others continued conservative treatment, due to nonradical resection or contraindications to surgery, with different efficacy.

Five acromegaly patients were excluded from the study because of moderate mitral regurgitation (4 points) and congenital atrial septal defect, with hemodynamic consequences like increased right ventricle systolic pressure (RVSP).

The characteristics of the analyzed group were as follows:

- 108 patients qualified for surgery. In 57 patients, the operation was considered radical, and therefore, they were considered cured from acromegaly. Thus, conservative treatment was not continued. In 51 patients, the surgery was not radical and treatment was continued (octreotide LAR, lanreotide autogel, pasireotide LAR, and pegvisomant) [9, 10].
- Fourteen patients had contraindications to surgical treatment, due to general condition or location of the pituitary gland tumour.
- In six patients, the operation was postponed due to various clinical conditions, often related to hormonal disorders within different pituitary gland axes (mostly thyrotropic), which required treatment.
- Information regarding surgeries in 12 remaining individuals was unavailable.

Majority of the 140 analyzed patients were diagnosed with sporadic pituitary gland adenoma. In five patients, the symptoms of acromegaly resulted from rare syndromes, such as

- familial isolated pituitary adenoma (FIPA)—two cases [11, 12];
- Carney complex, comprising, among others, heart and cutaneous myxomas and pituitary gland adenoma—one case [13];
- ectopic acromegaly due to bronchial neuroendocrine tumour secreting growth hormone-releasing hormone (GH-RH)—one case [7];
- MEN1, characterized by multiple endocrine tumours—one case [1, 10, 14].

Six patients died during the follow-up. Mortality causes included glioblastoma (one patient), alcoholic liver cirrhosis (one patient), central nervous system bleeding caused by aneurysm rupture (two patients), and cerebral ischemic infarction (one patient).

We did not determine one cause of death, because there was no contact with the patient's family.

Information regarding survival of 12 patients was not available. The average follow-up time amounted to 4 years and 130 days.

### 2.2. Diagnostic Methods

Patients were diagnosed at the Department of Endocrinology of the Medical Centre for Postgraduate Education, based on clinical symptoms of acromegaly, as well as results of testing and medical imaging.

The diagnostic criteria included the following:

- Typical acromegalic features. There were often used old photos of the patient (5–20 years before), to estimate the onset of symptoms prior to diagnosis [1, 2, 15].
- Lack of GH inhibition (<1.0 *μ*g/L) in the oral 75 g glucose tolerance test (OGTT). This method was used in recently diagnosed patients and patients following their first adenoma surgery to assess treatment effectiveness. In patients treated with somatostatin analogues, GH was tested casually. The reference value was the same as for the oral glucose tolerance test. GH was tested using the immunochemiluminescence method, with the IMMULITE 2000 analyser (Siemens, Los Angeles, California, USA) or LIAISON XL analyser (DiaSorin, Italy).
- Increased level of IGF-1 in comparison to the normal values for age and sex. IGF-1 was tested using the immunochemiluminescence method, with the IMMULITE 2000 analyser (Siemens, Los Angeles, California, USA) or LIAISON XL analyser (DiaSorin, Italy).
- MRI of the central nervous system, with evaluation of the pituitary gland.
- Arterial hypertension and glucose metabolism disorders (diabetes, prediabetes) were diagnosed according to the guidelines of the Polish Arterial Hypertension Society [16] and Polish Diabetic Society [17].

### 2.3. Echocardiographic Examination

Echocardiography was performed using the Vivid 4 (Haifa, Israel) device before 2012 and Vivid 9 (Horten, Norway) from 2012 onwards. A sector transducer with a frequency of 3.2 MHz was used. The following data were recorded:

- Left ventricular diastolic dimension and myocardial thickness: interventricular septum (IVS), posterior wall (PW), and left ventricular mass (LVM). Measurements were conducted in 2D imaging. LVM was calculated using the formula of the American Society of Echocardiography (ASA) modified by Devereux et al. [18]. LVM was indexed in accordance with body surface area (BSA) to obtain the LVMi value. LV hypertrophy was defined as an LVMi of >115 g/m^2^ in men and >95 g/m^2^ in women.
- Diastolic function parameters [19, 20]:
  - E'med. (cm/s)—early diastolic velocity of the septal mitral annulus assessed with tissue Doppler exam;
  - E/E'—mitral E-wave velocity divided by mitral annular velocity;
  - E/A—mitral E-wave velocity divided by mitral A-wave velocity.
- Left atrial volume index (LAVI), measured using standard 4-chamber projection and the curvature of the diastolic surface area of the left atrium. The software calculated the value.
- Systolic function parameters:

Ejection fraction (EF) was calculated according to the Simpson formula. The limitations of this method are caused by difficulty in planimetric endocardial silhouette layering in poorly visualized projections. Accordingly, in our study in the acromegaly patients with imaging difficulties, the visual estimation method was used, as described in the literature [21, 22]. EF was assessed by two independent specialists in echocardiography. According to the up-to-date guidelines, the low-normal limit was set at EF = 55%. Due to the fact that the EF values have no practical application when they are within normal limits (≥55%) and that many variables affect this parameter (including, among others, hydration and heart rate), in both groups, the EF value was not recorded unless the value fell below 55% (i.e., abnormal EF).

(v) Global longitudinal strain (GLS) calculated using 2-dimensional speckle tracking. This parameter was assessed in patients examined using the Vivid 9 device.
(vi) Right ventricular (RV) morphologic and functional parameters:

The size of RV was assessed with four-chamber apical projection due to higher reliability and repeatability of the results compared with other methods. The analysis included the transverse basilar measurement, with a normal value of ≤41 mm [23]. The thickness of the free wall of the right ventricle was measured with substernal projection.

Right ventricle systolic pressure (RVSP) was estimated as an indicator of right ventricle function in patients with tricuspid regurgitation. Mild tricuspid regurgitation is present in many healthy individuals, with RVSP estimates at below 35 mmHg considered normal [23]. Considering the lack of practical value of RVSP when it remains within normal limits, and the elevated error rate when mild tricuspid regurgitation is assessed, we only recorded abnormal RVSP (above 35 mmHg) values.

(vii) Diameter of thoracic aorta:

This measurement was performed one intercostal space above the routine long sternal projection (LAX).

All these results were retrospectively compared with results of 52 healthy individuals constituting the control group.

### 2.4. Statistical Analysis

Normal distribution was confirmed with the Shapiro–Wilk test before data was analyzed. Depending on the distribution of variables, appropriate tests were used for further analysis. Student's *t*-test was used for comparison of average values of two variables with normal distribution (for paired data or depending on the situation). We used nonparametric analysis when the null hypothesis of normal variable distribution was rejected. The median was compared with the Mann–Whitney *U* test. The chi-squared test was used for comparison of the basic clinical characteristics between the groups. Statistical analyses were performed with a significance level of 5% (*p* value < 0.05). The statistical analysis was carried out with the STATISTICA 12.5 software.

### 2.5. Ethics Statement

The Bioethics Committee, Medical University of Warsaw, approved the retrospective analysis of data obtained from echocardiograms of patients with acromegaly and testing of the healthy individuals representing the control group.

## 3. Results

Demographics of the studied group and control group, clinical characteristics, and average GH and IGF-1 levels measured in patients suffering from acromegaly are presented in Table 1.

No statistically significant difference was detected in age, sex, and BSA between controls and patients with acromegaly. Patients with acromegaly presented with higher BMI, and this group had an increased prevalence of arterial hypertension and glucose metabolism disorders such as diabetes and prediabetes. Results of the evaluated echocardiographic parameters are presented in Table 2.

There were no statistically significant differences in the size of the left and right ventricles between both groups. The thickness of the right ventricular free wall did not differ between the cohorts. Only two individuals out of 140 patients with acromegaly presented with an EF below 55% (50% and 52%). According to the up-to-date guidelines of ASE and EACVI, an EF below 52% in men should be interpreted as a lower normal limit [23]. Thus, only one patient from the analyzed group presented with an abnormal EF, indicating mild systolic dysfunction. Statistically significant differences between patients and controls were detected in the left atrial size (LAVi, 41.4 mL/m^2^ versus 29.9 mL/m^2^, *p* < 0.001), left ventricular myocardial thickness (IVS, 13.0 mm versus 10.0 mm, *p* < 0.001; PW, 12.0 versus 10.0, *p* < 0.001), and its derivative, the left ventricular myocardial mass (LVMi, 133.5 g/m^2^ versus 97.0 g/m^2^, *p* < 0.001), as well as diastolic function measured with E'med. (6.5 cm/s versus 9.0 cm/s, *p* < 0.001), E/E' (10.0 versus 8.0, *p* < 0.001), and E/A (0.9 versus 1.2, *p* < 0.001). In the evaluation of systolic function using GLS, we recorded statistically significant differences (−19.2% versus −20.7%, *p* < 0.01), as well as in the evaluation of ascending aorta diameter (Ao, 33 mm versus 31 mm, *p* < 0.01). When this parameter was normalized to BSA, the difference was not statistically significant (Ao, 16.8 mm/m^2^ versus 15.5 mm/m^2^, NS). Significantly more patients presented with abnormal RVSP compared to controls (18.5% versus 3.8%, *p* < 0.05).

## 4. Discussion

Recently, several articles have been published which reported that cardiovascular changes due to acromegaly are not so significant as researches claimed previously. It is an open question whether this is the result of more effective therapy or making diagnosis at the earlier stage of the disease or maybe the conclusions of the previous studies were exaggerated.

For instance, Silva et al. published a study conducted on 40 patients with active acromegaly, who were evaluated with echocardiography and cardiac magnetic resonance imaging with LVMI and EF assessment. The EF was normal in all patients, and only 5% were diagnosed with left ventricular hypertrophy. The examination was repeated after 12 months of treatment, but there were no statistically significant differences in the obtained results independently of the dynamics of the GH and IGF-1 values, indicating biochemical disease control [24]. Fazeli et al. expressed similar doubts regarding the influence of GH and IGF-1 on the cardiovascular system. They conducted a study on 23 patients with pituitary insufficiency regarding GH secretion. After 12 months of treatment, there were no differences in echocardiographic parameters between the groups (15 treated patients and 9 untreated patients) [25].

The findings of our study correspond with the conclusions of the abovementioned trials. The results indicate that there was no difference between controls and patients in left ventricle size, right ventricle size, and thickness of right ventricular free wall. Average values of the right and left ventricle sizes were within normal limits established by European and American guidance [23]. The thickness of the right ventricular free wall was similar in both groups and slightly exceeds the upper normal limit. Thus, the influence of GH and IGF-1 on the morphology of the right ventricle is in doubt.

Literature focusing on acromegaly and its influence on the cardiovascular system often states that this condition affects both right and left ventricles and emphasizes that both ventricle hypertrophies are a significant pathognomonic feature [5, 7, 26]. Data regarding the left ventricular hypertrophy assembled in our centre confirm these observations. However, there are only a few studies on right ventricular parameters, including the thickness of the right ventricular free wall. A study conducted by an Italian health centre reported abnormal thickness of the right ventricular free wall in patients with acromegaly [27, 28]. However, a small number of patients (*n* = 20) was a significant limitation of this study.

In 2015, a study from a health centre in Beijing analyzed 108 patients diagnosed with acromegaly. The echocardiographic parameters of patients were compared with a control group. The diastolic right ventricular dimension was normal and comparable in both groups, as in our study [29]. However, in the analysis of RVSP, which is a functional parameter of the right ventricle, there is a significant difference between the two groups. Abnormal values of RVSP were detected in 26 acromegaly patients, which constituted 18.5% of this group.

The reason of increased RVSP in acromegalic patients is not known, but probably, it is related to obstructive sleep apnea, which occurs significantly more commonly in these individuals [30].

The assessment of the left ventricular systolic function, which was normal in all patients except one with a mild dysfunction, was supported with measurement of the left ventricular global longitudinal strain (GLS) [31, 32]. Its new parameter in echocardiography, which detects subclinical cardiac involvement in different systemic diseases while systolic function is assessed by EF, is normal [33–35]. Up-to-date echocardiography experts established that the limit of normal value for this parameter is −20% [23]. In our study, acromegalic patients had GLS slightly below normal value and it was statistically significant different in comparison to the control group. Different results of GLS in acromegalic patients were reached by Volschan et al. [36]. Because studied groups in both trials were not homogenous, hard conclusions cannot be drawn. Further studies using GLS and assessment of the potentially positive impact of treatment on this parameter are necessary.

There are few reports on large vessel characteristics, including thoracic aorta, in acromegaly [37]. In our study, we measured the thoracic aorta diameter in 114 patients with acromegaly and in all controls. Abnormalities were detected in both groups. The largest diameter of the aorta measured in the analyzed group of patients amounted to 56 mm, whereas in controls, a value was noted (43 mm). Considering the average absolute values, there was a noticeable difference between the groups. However, when the parameter was normalized to BSA, this difference was not statistically significant. In both groups, the average/normalized dimension of the thoracic aorta did not exceed the accepted normal value of 15 ± 2 mm/m^2^ [23].

The most significant differences between the groups were recorded in the left atrium size, left ventricular mass, and diastolic function expressed as E'med., E/E', and E/A.

Just the left ventricular hypertrophy and impaired diastolic function are the most common reported changes in acromegalic patients. Similar results come from Beijing, where 108 patients were examined, and what is interesting, BMI and age were positively correlated with both hypertrophy and diastolic dysfunction, and just these factors, not GH and IGF-1, were the most important predictors for hypertrophy [29].

It is unclear if the increased left atrial volume results directly from left ventricular hypertrophy and impaired diastolic function or the increased expression of GH receptors in the cardiomyocytes of the left atrium. Interestingly, more than 38% of acromegaly patients were found to have normal E'med., which is a very good and stable indicator for diastolic function, and about 62% of these patients was diagnosed with an enlarged left atrium. Similar findings are noted in athletes for whom the enlargement of the left atrium is a normal adaptive mechanism. It is possible that GH plays a similar role in physical exercise or physical exercise stimulates GH secretion. This is an interesting issue for further studies [38, 39].

It is worth remembering that an enlarged left atrium increases the risk of arrhythmia, especially atrial fibrillation. This suggests that there is a need for periodic follow-up using a Holter ECG monitor, as these patients are predisposed to paroxysmal arrhythmia. Additionally, thyrotrophic secretion disorders, which often accompany acromegaly, may have an unfavorable impact.

### 4.1. Limitations

Patients with acromegaly are more difficult to examine by means of echocardiography due to a higher BMI and bone remodelling, presenting with costosternal cartilage hypertrophy, and significant thickening of the ribs, which narrows the intercostal space. Therefore, in some patients, it was not possible to perform a complete examination and to satisfactorily register all evaluated parameters.

Furthermore, the study is also limited by the fact that, until 2012, examinations were performed using the Vivid 4 echocardiograph, which had lower technical capabilities than the Vivid 9 device. The Vivid 9 device was used for the majority of patients and all controls. The lack of GH and IGF-1 testing in the controls also limits the study. Testing was not performed because the controls did not reveal any symptoms suggesting acromegaly.

In addition, certain medical information for some patients were not available (7 patients—no information regarding concomitant diseases, 12 patients—no information regarding past surgeries). This is because data from before 2013 are not available in electronic form, and there is no national registry of patients suffering from acromegaly.

The restriction of testing two echocardiographic parameters, ejection fraction and right ventricular systolic pressure, may also be considered a limitation. There are no numerical values for all the parameters in all controls and patients; therefore, it is not possible to perform a complete statistical analysis. However, setting the normal limiting value as a cut-off point enabled a descriptive analysis to be performed.

## 5. Conclusions

There is no systolic dysfunction in acromegalic patients regardless of the degree of disease activity. Although we found subclinical systolic dysfunction in our study group, it is too early to draw final conclusions. Further studies are required to assess GLS in homogenous patients before treatment. More frequent occurrence of left ventricular hypertrophy and deterioration of diastolic function in acromegalic patients may result from comorbidity and other factors like BMI and may not be directly related to elevated GH and IGF-1 values.

The left atrium is enlarged more often in acromegalic patients, regardless of diastolic dysfunction. This requires more attention from clinicians since the enlarged atrium may increase the risk of arrhythmias, including atrial fibrillation. Moreover, the studied patients did not present morphological changes in the right ventricle, and there is no statistically significant difference in the indexed measurement of thoracic aorta in patients compared to the control group.

## Figures and Tables

**Table 1:** Demographic, clinical, and biochemical features of patients with acromegaly versus the control group.

	Patients with acromegaly	Control group	*p* value
*N* = total (♀/♂)	140 (91/49)	52 (27/25)	0.10
Age, years	50.5 ± 13.8	47.4 ± 12.3	0.16
BMI, kg/m^2^	30.1 ± 5.2	27.9 ± 4.6	<0.01
BSA, m^2^	2.0 ± 0.2	1.9 ± 0.2	0.24
HT, yes/no	81/52	17/35	0.001
DM, yes/no/pre-DM	35/52/46	0/49/3	0.001
GH, μg/L	4.30 (0.05–79.20)	—	—
IGF-1, μg/L	705.3 ± 388.6	—	—

Abbreviations: BMI: body mass index; BSA: body surface area; DM: diabetes mellitus; GH: growth hormone; HT: hypertension; IGF-1: insulin-like growth factor-1; pre-DM: prediabetes; GH: growth hormone.

**Table 2:** Echocardiography parameters in patients with acromegaly versus the control group.

	Patients with acromegaly [*N*]	Control group [*N*]	*p* value
Cardiac chamber size			
LVEDd, mm	48.0 (24.0–63.0) [95]	47.0 (39.0–58.0) [52]	NS (0.07)
RVd-4CH, mm	35.0 (24.0–53.0) [132]	34.5 (26.0–47.0) [52]	NS (0.61)
LAVI, mL/m^2^	41.4 (19.5–124.0) [133]	29.9 (15.9–53.3) [52]	< 0.001
LV/RV thickness LV mass			
IVS, mm	13.0 (8.0–19.0) [139]	10.0 (7.0–15.0) [52]	< 0.001
PW, mm	12.0 (7.0–18.0) [136]	10.0 (7.0–14.0) [52]	< 0.001
LVMI, g/m^2^	133.5 (69.0–252.0) [140]	97.0 (44.0–163.0) [52]	< 0.001
RV free wall thickness, mm	6.0 (3.0–10.0) [130]	6.0 (4.0–10.0) [52]	NS (0.75)
Diastolic function			
E'med., cm/s	6.5 (3.0–15.0) [140]	9.0 (5.0–18.0) [52]	< 0.001
E/E'	10.0 (5.0–27.0) [140]	8.0 (5.0–13.0) [52]	< 0.001
E/A	0.9 (0.5–1.8) [135]	1.2 (0.5–2.3) [52]	< 0.001
Systolic function			
EF, % < 55, *n*	2/140	0/52	
GLS, %	19.2 (8.4–27.1) [81]	20.7 (14.6–30.2) [51]	< 0.01
Ascending aorta			
Ao asc., mm	33.0 (24.0–56.0) [114]	31.0 (25.0–43.0) [52]	< 0.01
Ao asc.index, mm/m^2^	16.8 (12.2–25.3) [114]	15.5 (12.9–21.4) [52]	NS (0.05)
RVSP, mmHg > 35, *n*	26/140	2/52	< 0.05

Abbreviations: Ao asc.: ascending aorta; Ao asc.index: ascending aorta index; E/A: mitral E-wave velocity divided by mitral A-wave velocity; E/E: mitral E-wave velocity divided by mitral annular velocity; E'med.: early diastolic velocity of the septal mitral annulus assessed with tissue Doppler exam; EF: ejection fraction; GLS: global longitudinal strain; IVS: interventricular septal; LVEDd: left ventricular end-diastolic diameter; LAVI: left atrial volume index; LVMI: left ventricule mass index; PW: posterior wall; RVd-4CH: right ventricular dimension—4 chamber projection; RVSP: right ventricular systolic pressure.
